# Alternative Lengthening of Telomeres and Differential Expression of Endocrine Transcription Factors Distinguish Metastatic and Non-metastatic Insulinomas

**DOI:** 10.1007/s12022-020-09611-8

**Published:** 2020-02-26

**Authors:** Wenzel M. Hackeng, Willemien Schelhaas, Folkert H. M. Morsink, Charlotte M. Heidsma, Susanne van Eeden, Gerlof D. Valk, Menno R. Vriens, Christopher M. Heaphy, Els J. M. Nieveen van Dijkum, G. Johan A. Offerhaus, Koen M. A. Dreijerink, Lodewijk A. A. Brosens

**Affiliations:** 1 Department of Pathology, University Medical Center Utrecht, Heidelberglaan 100, 3584 CX Utrecht, The Netherlands; 2 Department of Pathology, Amsterdam University Medical Center, Amsterdam, The Netherlands; 3 Department of Surgery, Amsterdam University Medical Center, Amsterdam, The Netherlands; 4 Department of Endocrinology and Internal Medicine, University Medical Center Utrecht, Utrecht, The Netherlands; 5 Department of Surgery, University Medical Center Utrecht, Utrecht, The Netherlands; 6 Department of Pathology, Johns Hopkins Medical Institutions, Baltimore, USA; 7 Department of Endocrinology and Internal Medicine, Amsterdam University Medical Center, Amsterdam, The Netherlands

**Keywords:** Pancreatic neuroendocrine tumor, Insulinoma, Malignant insulinoma, Liver metastasis, Neuroendocrine cells

## Abstract

Insulin-producing pancreatic neuroendocrine tumors (PanNETs)/insulinomas are generally considered to be indolent tumors with an excellent prognosis after complete resection. However, some insulinomas have a poor prognosis due to relapses and metastatic disease. Recently, studies in non-functional PanNETs indicated that behavior can be stratified according to alpha- and beta-cell differentiation, as defined by expression of the transcription factors ARX and PDX1, respectively. It is unknown whether similar mechanisms play a role in insulinomas. Therefore, we determined ARX and PDX1 expression in a cohort of 35 sporadic primary insulinomas and two liver metastases of inoperable primary insulinomas. In addition, WHO grade and loss of ATRX or DAXX were determined by immunohistochemistry, and alternative lengthening of telomeres (ALT) and *CDKN2A* status by fluorescence in situ hybridization. These findings were correlated with tumor characteristics and clinical follow-up data. In total, five out of 37 insulinoma patients developed metastatic disease. Metastatic insulinomas were all larger than 3 cm, whereas the indolent insulinomas were smaller (*p* value < 0.05). All three primary insulinomas that metastasized showed ARX expression, 2/3 showed ALT, and 1/3 had a homozygous deletion of *CDKN2A* as opposed to absence of ARX expression, ALT, or *CDKN2A* deletions in the 32 non-metastatic cases. The two liver metastases also showed ARX expression and ALT (2/2). The presence of ARX expression, which is usually absent in beta-cells, and genetic alterations not seen in indolent insulinomas strongly suggest a distinct tumorigenic mechanism in malignant insulinomas, with similarities to non-functional PanNETs. These observations may inform future follow-up strategies after insulinoma surgery.

## Introduction

Insulinomas are the most common functional pancreatic neuroendocrine tumor (PanNET) type and are diagnosed by the triad of hypoglycemic symptoms, low blood glucose concentrations, and relief of symptoms after glucose administration. Most insulinomas are indolent tumors: there are few mitoses (low grade) and metastases are very rare [1, 2]. In contrast, 40–50% of non-functional PanNETs present with liver metastases at time of initial diagnosis [3, 4]. Surgery for insulinomas is primarily indicated to alleviate symptoms of hypoglycemia. Survival after surgery is not different from the general population [5, 6]. There are no insulinoma-specific international recommendations for follow-up after surgery [5, 7]. Nevertheless, about 10% of insulinoma patients develop metastases that are mostly present at time of initial diagnosis, but may sometimes develop years after resection of the primary tumor [8]. Median survival is less than 2 years in patients with metastatic insulinoma, similar to metastatic non-functional PanNET [9–11].

Because of the rarity of metastatic insulinomas, little is known about the mechanisms of tumorigenesis. Recent sequencing studies identified recurrent *YY1* gene mutations in insulinomas; however, relapses or metastases were rarely reported in the studied cohorts [12–15]. There is a need to increase our understanding of insulinoma development, in particular of metastatic insulinomas. This will improve identification of patients at risk for recurrence, who may benefit from follow-up after surgery and thereby earlier detection and treatment of metastases.

Metastatic non-functional PanNETs are more common and have been characterized more extensively. Next-generation sequencing studies have demonstrated that sporadic PanNETs harbor relatively few gene mutations. The genes that are most frequently mutated are *MEN1*, *ATRX*, and *DAXX* [12, 16, 17]. Mutually exclusive mutations in *ATRX* or *DAXX*—coding for two chromatin-modifying proteins which form a histone chaperone complex—are associated with the alternative lengthening of telomere phenotype (ALT) [18]. Immunohistochemical loss of ATRX and DAXX can be used as a surrogate marker of inactivating mutations of *ATRX* or *DAXX*, respectively [18, 19]. The presence of either of these alterations is associated with recurrence and liver metastases [20–25]. In addition to *ATRX* or *DAXX* mutations and ALT, loss of ARID1A, loss of H3K36 trimethylation (H3K36me3) by *SETD2* dysfunction, and *CDKN2A* deletions were also reported to be of prognostic value for non-functional PanNET [26]. A recent finding in non-functional PanNETs is that alpha- or beta-cell types-of-origin may predict clinical behavior [20, 27]. Beta-cell like non-functional PanNETs, marked by expression of the endocrine transcription factor PDX1, were generally indolent, while almost all relapses were observed in the group of alpha-cell like non-functional PanNETs marked by ARX expression. Furthermore, somatic mutations in *ATRX*, *DAXX*, and *MEN1* and acquisition of ALT were observed more often in the alpha-type non-functional PanNETs [20, 27].

Whether the ARX and PDX1 transcription factors play a role in the clinical behavior of insulinomas is not known. We determined protein expression of ARX and PDX1 together with ATRX, DAXX, ARID1A, and H3K36me3 by immunohistochemistry, as well as ALT and *CDKN2A* deletions by fluorescence in situ hybridization (FISH), in a cohort of clinically defined sporadic insulinomas.

## Materials and Methods

### Study Cohort

The study was approved by the University Medical Center (UMC) Utrecht Biobank Research Ethics Committee. Tissue-microarrays (TMAs) were constructed of primary sporadic insulinomas resected between 1991–2017 and 1997–2017 for the UMC Utrecht and Amsterdam UMC, respectively. Inherited cases (e.g., MEN1 syndrome) were excluded. Neuroendocrine tumor diagnosis was confirmed by an experienced gastrointestinal pathologist (LAAB). Three 0.6-mm cores per tumor were randomly taken from annotated tumor areas in formalin-fixed paraffin-embedded (FFPE) blocks. In case of multiple tumors, the largest tumor was used. Biopsies of insulinoma liver metastases were identified by a search in the UMC Utrecht pathology archive.

Information on age, sex, multifocality, surgery type, surgery date, tumor size, location, grade, resection margin, and lymph nodes was collected from pathology reports. If possible, macroscopic tumor size was used. Free margins were interpreted as R0, also if the distance was less than 1 mm from the resection margin. Medical records were reviewed to collect information on the functional status of the tumor, presence of genetic syndromes, and follow-up. Events of tumor relapse (local recurrence, liver metastases, or other metastases) were either histologically proven or diagnosed by the treating clinician. The first radiological evidence of proven relapse was used as event time point. Follow-up time is counted from date of surgery until described events, death, or was censored at the last visit to a relevant hospital clinician (surgery, internal medicine, endocrinology, gastroenterology, or oncology) or most recent clinic visit. For overall survival, any cause of death and the most recent clinic visit were used. Relapse was defined as any distant metastasis (liver or other location) or local recurrence. Relapse-free, distant-free, and liver metastases-free survival were censored at last visit to a relevant hospital clinician.

### Immunohistochemistry

Four-micrometer sections of FFPE tissue were cleared at 60 °C and deparaffinized in xylene. Endogenous peroxidase was blocked by immersion in 0.6% H_2_O_2_ (7210, Merck, Kenilworth, USA) in methanol for 15 min. Antigen retrieval was performed by cooking slides in a 10 mM citrate (pH 6) or 10/1 mM Tris/EDTA (pH 9) solution for 20 min. Nonspecific binding was reduced by with Protein Block Serum Free (X0909, Dako, Santa Clara, United States of America). Antibodies were diluted in normal antibody diluent (Immunologic, Duiven, The Netherlands) and applied on the slides (Table 1). After incubation of post antibody blocking solution for 15 min (Immunologic), the secondary antibody Poly-HRP-goat anti Mouse/Rabbit IgG (cat. no. VWRKDPVB110HRP, Immunologic) was incubated for 30 min. Peroxidase activity was detected by DAB (D5637, Sigma, St. Louis, USA) or Bright-DAB (cat. no. VWRKBS04–110, Immunologic) as chromogen for 8 min. After all incubation steps, except the protein block, slides were washed with PBS-Tween-20 0.1% four times. Slides were counterstained with hematoxylin and mounted with Pertex (Histolab, Askim, Sweden).

**Table 1:** Antibodies and protocol variations

Antibody target	Company	Name	Species and (clone)	Pre-treatment	Dilution/time/temperature	Substrate	Scoring method
DAXX	Atlas antibodies, Bromma, Sweden	HPA008736	Rabbit PAB	ARS/pH6 20 min	1:100 1 h RT	Bright-DAB	Negative if positive nuclear staining < 5% of tumor cells
ATRX	Sigma, St. Louis, MO	HPA0001906	Rabbit PAB	ARS/pH9 20 min	1:400 overnight 4 °C	DAB	Negative if positive nuclear staining < 5% of tumor cells
ARX	Millipore, Burlington, MA	MABN102	Mouse MAB (11F6.2)	ARS/pH6 20 min	1:2000 1 h RT	DAB	Positive if weak nuclear staining > 50% or intermediate/strong nuclear staining > 10% of tumor cells
PDX1	Abcam, Cambridge, UK	ab134150	Rabbit MAB (EPR3358(2))	ARS/pH6 20 min	1:2000 1 h RT	DAB	Positive if weak nuclear staining > 50% or intermediate/strong nuclear staining > 10% of tumor cells
Ki67	Immunologic, Duiven, The Netherlands	VWRKILM9252-C05	Mouse MAB (MIB1)	ARS/pH6 20 min	1:200 1 h RT	DAB	Digital image analysis of nuclear expression in at least 2000 tumor cells
Glucagon	Cell Marque, Rocklin, CA	259A-15	Rabbit PAB	ARS/pH6 20 min	1:100 1 h RT	Bright-DAB	Positive if cytoplasmic staining > 10% of tumor cells, scattered if < 10% of tumor cells
Insulin	Dako, Santa Clara, CA	A564	Rabbit PAB	ARS/pH6 20 min	1:100 1 h RT	DAB	Positive if cytoplasmic staining > 10% of tumor cells, scattered if < 10% of tumor cells
H3K36me3	Abcam, Cambridge, UK	ab9050	Rabbit PAB	ARS/pH6 20 min	1:2000 1 h RT	DAB	Negative if positive nuclear staining < 30% of tumor cells
ARID1A	Abcam, Cambridge, UK	ab182560	Rabbit MAB (EPR13501)	ARS/pH6 20 min	1:1000 1 h RT	DAB	Negative if positive nuclear staining < 5% of tumor cells

*MAB* monoclonal antibody, *PAB* polyclonal antibody, *ARS* antigen retrieval solution, *RT* room temperature, *DAB* 3,3'-Diaminobenzidine

Scoring was performed by at least two independent researchers (WMH, WS, LAAB), blinded for each other’s results and clinical information. Disagreements were resolved by consensus.

For ARX and PDX1, negative protein expression in tumor tissue was defined as weak nuclear staining in < 50% of cells or strong nuclear staining in < 10% of cells. Positive expression was defined as weak nuclear staining in > 50% of cells or intermediate/strong nuclear staining > 10% of cells [27]. For insulin and glucagon, cytoplasmic staining of > 10% of cells was considered positive expression for the respective peptide hormone. Normal islets, containing a mix of cells expressing or not expressing the respective peptide hormone, were used as positive and negative controls, respectively. If < 10% of cells had expression, cases were called scattered. DAXX, ATRX, and ARID1A were considered negative if < 5% of cells had positive nuclear staining and if there was non-tumoral tissue present with positive nuclear staining serving as internal control, e.g., islets of Langerhans, stromal cells, endothelial cells, or lymphocytes [26, 28]. All negative cases in the TMA were also stained on whole sections to confirm the results. For H3K36me3 loss, a cut-off of 30% of cells was used [26, 29]. Negative cases without a positive internal control were non-informative. All cytoplasmic staining was ignored.

Ki67 labeling index (LI) was counted in at least 2000 cells by digital image analysis with Sectra (PACS, Sectra AB, Linköping, Sweden), as previously described [30]. Digital counts were confirmed by visual assessment. PanNETs were graded by the 2017 WHO criteria (Ki67 G1 < 3%, G2 3 to 20%, G3 > 20%) [31]. If the pathology report also mentioned tumor grade based on Ki67 or mitoses per 10 HPF, the highest grade was used for further analysis as the location of tumor cores not always represents the most proliferative region.

### Fluorescence In Situ Hybridization

After deparaffinization in xylene, 4 μm FFPE sections for *CDKN2A*/CEN9 FISH were pre-treated in 0.2 N HCL for 20 min, cooked in a 10 mM citrate buffer (pH 6) for 20 min and washed in PBS. Slides were then digested in proteinase K buffer for 10 min at 37 °C (5 μM Tris–HCL, 1μM EDTA, 1 μM NaCl, 10 mg/L Proteinase K), washed with PBS and dried. Ten microliters of *CDKN2A*/CEN9 probe mix (*CDKN2A/CEN* 9 Dual Color probe, Zytolight, Bremerhaven, Germany) was applied per slide. Slides were denatured at 78 °C for 5 min and cooled on ice for 5 min. Hybridization was performed in a ThermoBrite (Abbott Laboratories, Chicago, IL) at 37 °C overnight. After removing coverslips, slides were washed in washing buffer (WB) 1 (0.4× SCC, 0.5% NP-40, 73 °C), WB 2 (2× SCC, 0.1% NP-40, room temperature), WB 3 (2× SCC, room temperature), and PBS, for 2, 1, 5 min, and 20 s respectively. Nuclei were counterstained and mounted with Vectashield with DAPI (H-1200, Vector laboratories, Amsterdam, The Netherlands).

Slides for telomere/centromere FISH were cooked in 10 mM citrate buffer (pH 6) for 20 min, washed in dH_2_O and dried. Probes (TelC-Cy3 F1002 PNA 180723PL-01, Cent-FITC, F3013 172865, Panagene, Daejeon, Republic of Korea) were diluted in hybridization mix (50% deionized Formamide, 50% SCC 4×, 5% Dextran sulphate, Tween-20 0.5%) at a 400 nM concentration and applied on the slides. After 5 min denaturation at 84 °C, slides were cooled on ice for 5 min before hybridizing at 37 °C overnight. After removing coverslips, slides were washed in two cycles of 1× WB (70% Formamide, 30% dH2O, 10 mM Tris, 15 min) and 3× PBS (2 min each time). Nuclei were counterstained with DAPI in PBS 2 μg/ml (Sigma-Aldrich, D9542), and coverslips were mounted with Vectashield (H-1000, Vector laboratories).

Slides were stored at 4 °C before imaging, and viewed with a Leica DM5500 B using appropriate excitation and emission filters. Images were made at 100× magnification with a Z stack of 14 steps in Leica application Suite X (Leica Microsystems, Rijswijk, The Netherlands).

The number of *CDKN2A* gene probe and centromere probe signals were counted in at least 50 tumor cells for each case (WMH, WS). At least nine photographs of tumor tissue (confirmed on H&E) were made for counting of cells. Only intact non-overlapping nuclei with at least one centromere probe were counted. Multiple signals separated ≤ 1 signal distance were counted as one. If no signals were observed (gene and centromere) in the tumor and surrounding stromal cells or if there was too much background, cases were called non-informative. Homozygous loss was defined as at least 20% of counted cells lacking *CDKN2A* probe signals with at least one CEP9 probe. Hemizygous deletion of *CDKN2A* was concluded if 45% of counted cells had one *CDKN2A* probe and two CEP9 probes; monosomy of chromosome 9 (which can be considered hemizygous loss) if 15% of cells had one *CDKN2A* probe and one CEP9 probe. Cut-off values were based on previous literature [26, 32]. If results were discordant, additional photos were made and at least 100 cells were counted (WMH).

ALT positivity was defined as ultra-bright, intra-nuclear telomere FISH signals, 10× the signal intensity of cumulative single telomere sum intensities in normal stromal/endothelial cells, which are present in more than 1% of cells [18, 33]. The percentage of ALT cells > 1% was determined on × 20 magnification by visual assessment (WMH) in areas of tumor tissue (annotated in H&E slide), with a cut-off of more than 20 ultra-bright foci per tumor core (max 2000 cells). If less than 1% by visual assessment, all ultra-bright foci were counted. All ultra-bright foci were confirmed at × 100 magnification. Using the same laser/microscope settings, representative ultra-bright foci near stromal cells were photographed and digital grayscale TIFF images exported for each fluorophore channel. Telomere signals were quantified using Telometer (a free custom software ImageJ plug-in, downloaded from demarzolab.pathology.jhmi.edu/telometer). Rolling ball radius was set at the maximal telomeric signal diameter, which was measured per photograph. Signals were separated by the draw function if necessary. Centromere signals were used as positive hybridization efficiency control and to confirm specific probe binding.

### Statistics

Data was managed and statistical tests were performed in SPSS version 25 (IBM Nederland, Amsterdam, The Netherlands). The Kaplan Meier method was used to plot the effect of variables on events occurring over time, and significance was assessed by the log-rank test. The Χ^2^ or Fishers exact test was used when comparing categorical data between groups. Follow-up time was calculated using a reverse Kaplan Meier for relapse-free survival and observation time was calculated from date of surgery until last visit to a relevant hospital clinician, irrespective of events. *P* values < 0.05 were considered significant. Data was visualized in R version 3.5.1 using packages Beeswarm and Survplot.

## Results

**Fig. 1:**
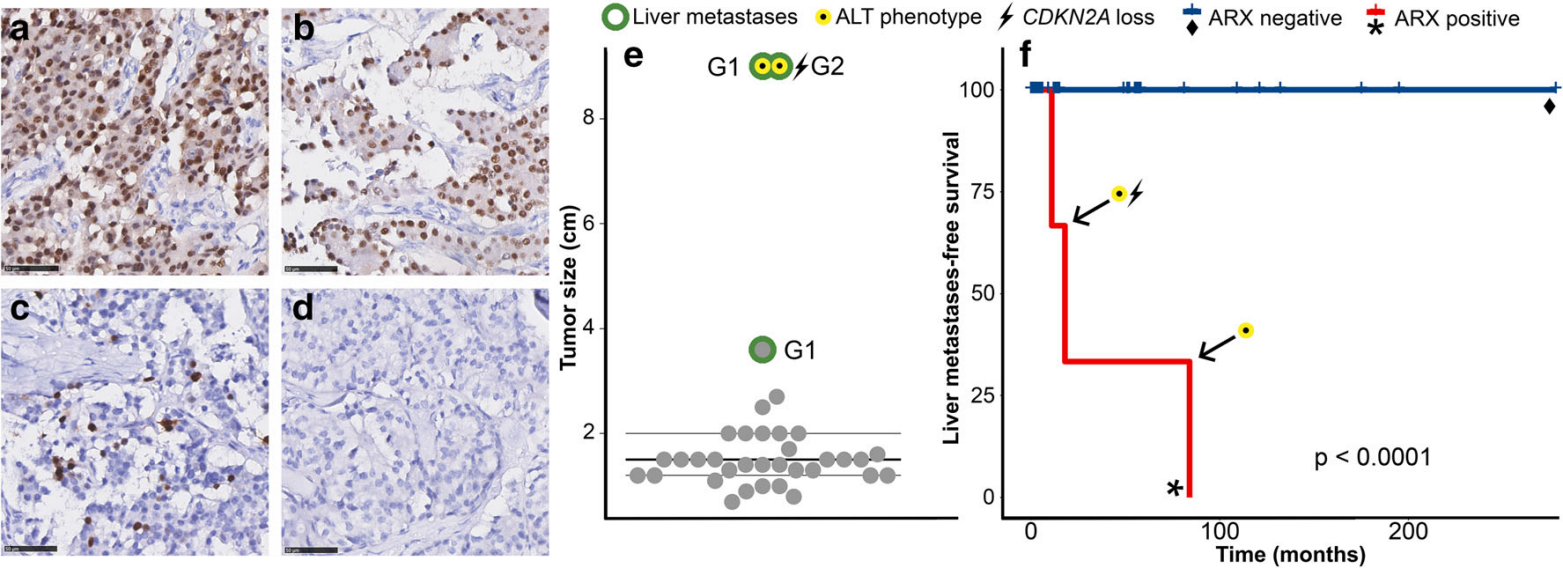
ARX expression, tumor size and liver metastases-free survival in primary insulinomas. **a**–**c** Positive ARX expression in the three metastatic primary insulinomas (patient 1, 2, 3) and no ARX expression in a non-metastatic insulinoma (**d**). Photos of immunohistochemistry, white-balanced, with 50-μm scale bar. **e** Tumor size plot. Cases with liver metastases in the size plot are circled (green), WHO grade is given. **f** Kaplan Meier of liver metastases-free survival for ARX expression in the primary insulinoma cohort. *P* value was calculated with the log-rank test

The cohort consisted of 35 primary insulinomas. The clinical characteristics of the patients are listed in Table 2. All cases were confirmed to be clinically functioning insulinomas by the treating hospital clinician. Three patients developed liver metastases during follow-up, one of whom also had local recurrence and tumor depositions around the uterine appendages. While all metastastic primary cases initially presented with symptomatic hypoglycemia, only two out of three cases had hypoglycemic events at relapse by liver metastasis (Table 3). Overall survival could not be analyzed as there were no deaths during follow-up. The metastatic primary insulinomas were larger than the indolent primary insulinomas, but this did not reach statistical significance (mean 32 indolent insulinomas 1.48 cm, SD 0.45, mean 3 metastatic insulinomas 7.20 cm, SD 3.12, *t* test *p* = 0.086; Fig. 1). In addition, biopsies of liver metastases of two patients with inoperable primary insulinoma were available (Table 3), as well as a biopsy of a corresponding liver metastasis of one of the primary insulinomas in the cohort (patient 2, Table 3). When the sizes of the primary tumors of the inoperable insulinoma patients were included, the five metastatic insulinomas were significantly larger than the indolent cases (mean 1.48 cm, SD 0.45, metastatic mean 6.16 cm, SD 2.63, *t* test *P* = 0.016). A size cut-off of 3 cm perfectly separated all metastatic from indolent insulinomas.

**Table 2:** Patient and tumor characteristics

		Insulinomas
Sex (%)	Male Female	16 (46%) 19 (54%)
Age at surgery	Mean (± SD)	55 (± 18)
Median follow-up Median follow-up	Reverse KM + (mean, IQR months) Observation time (mean, IQR months)	49 (57, 5–81) 30 (52, 5–58)
Relapse (liver/other/local)	Yes No	3 (9%) 32 (91%)
Liver metastases (%)	Yes No	3 (9%) 32 (91%)
Other distant metastasis (%)	Yes No	1 (3%) 34 (97%)
Local recurrence (%)	Yes No	1 (3%) 34 (97%)
Death of all causes (%)	No	35 (100%)
Location (%)	Head Corpus Tail Multifocal Unknown	4 (11%) 4 (11%) 18 (51%) 2 (6%) 7 (20%)
Tumor size	Mean (± SD)	1.97 (± 1.84)
≥ 2 cm (%)		10 (29%)
≥ 3 cm (%)		3 (9%)
Grade (%)	1 2 3 Missing	31 (89%) 3 (9%) 0 (0%) 1 (3%)
Resection margins (%)	Free Involved Unsure Not mentioned	17 (49%) 7 (20%) 8 (23%) 3 (9%)
Lymph nodes (%)	Free Involved Not mentioned	14 (40%) 0 (0%) 21 (60%)
ARX (%)	Positive Negative	3 (9%) 32 (7%)
PDX1 (%)	Positive Negative	34 (97%) 1 (3%)
ATRX/DAXX (%)	ATRX negative DAXX negative Both positive Missing	0 (0%) 1 (3%) 33 (94%) 1 (3%)
Telomeres	Alterative lengthening of telomeres Normal telomeres Missing	2 (6%) 31 (89%) 2 (6%)
CDKN2A	Normal Monosomal Homozygous loss Not interpretable Not tested	18 (51%) 1 (3%) 1 (3%) 11 (31%) 4
ARID1A and H3K36me3	Both positive Missing Not tested	30 (97%) 1 (3%) 4
Insulin (%)	Positive Scattered Negative Missing Not tested	28 (90%) 2 (7%) 0 (0%) 1 (3%) 4
Glucagon (%)	Positive Scattered Negative Missing Not tested	13 (42%) 5 (16%) 12 (39%) 1 (3%) 4

*SD* standard deviation, *KM* Kaplan Meier, *IQR* interquartile range

**Table 3:** Characteristics of metastatic insulinomas

Patient	Type	Clinically functioning at presentation	Age (year)	Size (cm)	WHO grade (Ki67 LI)	Multifocal primary (number)	Insulin and glucagon	*CDKN2A*	ALT, ATRX/DAXX	ARX (% cells, intensity)	PDX1 (% cells, intensity)	Metastases (months after surgery)	Clinically functioning at relapse
1	Primary	Yes	5	3.6	1 (2.9%)	Yes (2)	Positive and positive	Normal	No, positive	Positive (10–20%, strong)	Positive (90–100%, strong)	Liver (11)	Yes
2	Primary	Yes	67	9.0	1 (0.1%)	No	Scattered and scattered	NI	Yes, positive	Positive (90–100%, Intermediate)	Negative (1%, Strong)	Liver (84)	No
	Liver met.	–	74	9.0^a^	ND	No	ND and ND	ND	Yes, ND	Positive (50%, Intermediate)	Negative (0%)	–	–
3	Primary	Yes	47	9.0	2 (7.7%)	Yes (2)	Scattered and scattered	Deletion	Yes, DAXX loss	Positive (90–100%, strong)	Positive (70–80%, intermediate)	Liver and other (18)	Yes
4	Liver met.	Yes	64	4.7^a^	2 (10%)	No	Scattered and ND	ND	Yes, ND	Positive (90–100%, strong)	Positive (90–100%, strong)	Liver (at presentation)	–
5	Liver met.	Yes	69	4.5^a^	2 (15%)	No	Positive and ND	ND	Yes, ND	Positive (90–100%, Strong)	Positive (90–100%, Strong)	Liver (at presentation)	–

*ALT* alternative lengthening of telomeres, *NI* not interpretable, *ND* not determined, *Ki67 LI* Ki67 labeling index

^a^Size of primary tumor in pancreas

Positive or scattered insulin expression was observed in all insulinomas. Positive or scattered glucagon expression was seen in about 60% of insulinomas. PDX1 expression was observed in 34/35 primary insulinomas (97%), and 2/3 liver metastases; in contrast, only 3/35 (9%) of primary insulinomas were ARX positive, while all liver metastases were ARX positive (Fig. 1, Tables 2 and 3). All ARX positive primary insulinomas developed metastases during follow-up. The ARX-positive percentage of cells varied between 10 and 90% of cells (Fig. 1, Table 3), and PDX1 expression was intermediate to strong in 70–100% of cells for all positive cases. Two (of 3) metastasizing primary insulinomas were multifocal (two tumors per patient). To exclude the possibility of the smaller tumor being the insulinoma and the larger tumor (tested in the TMA) possibly being an ARX-positive non-functional PanNET, whole slides of all multifocal tumors were stained for ARX, PDX1, and insulin. ARX, PDX1, and insulin expression was identical between tumors of the same patient. Thus, strong ARX expression in more than 10% of cells identified metastatic insulinomas with a 100% sensitivity and specificity. Of note, one of the metastatic insulinomas showed areas with < 10% ARX positive cells with scattered positive cells when assessing the whole slide (Patient 1, Fig. 1c). Although no obvious heterogeneity was seen between different tumor cores when scoring for ARX and PDX1, it is conceivable that the number of ARX-positive cells was underestimated using TMA cores for other cases. We therefore sought to further confirm our results by reviewing all ARX-negative insulinomas by digital image analyses on the TMA similar to the Ki67 count [30]. In all these cases, expression was observed in far less than 10% of cells, most often absent or in less than 1% of cells.

Of 33 insulinomas interpretable by telomere FISH, two cases (6%) had ALT which both developed liver metastases during follow up (Fig. 1; Table 3). All tested liver metastasis biopsies had ALT. All insulinomas (*n* = 34) with tumor tissue present in the TMAs had retained ATRX expression, only one case had heterogenic DAXX loss (also ALT positive). Insulin, glucagon, ARID1A, H3K36me3 IHC, and *CDKN2A* FISH were only tested on 31 cases, as for the last four insulinoma cases, no unstained TMA slides were available. All cases (*n* = 30) with tumor tissue present in the TMA had retained H3K36me3 and ARID1A expression. One case with homozygous *CDKN2A* deletion and one case with monosomal *CDKN2A* were observed (of 21 interpretable cases; Table 3). The case with homozygous *CDKN2A* loss developed liver metastases. The metastatic primary insulinomas were grade 1 (2/3) or grade 2 (1/3), and both metastasis biopsies were grade 2 (Ki67 labeling index, Table 3).

## Discussion

All metastatic insulinomas in this cohort were larger than 3 cm. Strikingly, all metastatic insulinoma lesions showed ARX expression, which was not observed in any of the indolent primary insulinomas. Four out of five (80%) metastatic insulinomas had ALT—not reported before in insulinomas—while none of the indolent insulinomas showed ALT. Interestingly, the two recent studies that identified ARX as marker for PanNET relapse after surgery, also included non-metastatic insulinomas: Cejas et al. found no ARX immunohistochemical expression in 17 primary insulinomas [27] and Chan et al. reported ARX mRNA expression (alpha signature) in one out of three insulinomas. None of the insulinomas in these studies metastasized, but remarkably, the ARX-expressing case in the study of Chan et al. was also *ATRX* mutated and very large (8 cm), while all the negative cases were small (< 2cm) and *ATRX*, *DAXX*, *MEN1* wild type [20]. These results are in line with our observation that there is an association between size, ALT, and ARX expression in insulinomas. To our knowledge, ALT has not been described before in any insulinoma. Retention of ATRX/DAXX protein expression in one of the insulinomas with ALT may be explained by non-truncating mutations, translocations, or other underlying mutations causing the ALT phenotype [34]. In the literature, presence of somatic *ATRX* and *DAXX* mutations or protein loss detected by immunohistochemistry is uncommon in sporadic insulinoma—in contrast to non-functional PanNET [12–15, 26]. A recent large whole-genome sequencing study definitively established that insulinomas and non-functional PanNETs have distinct genetic underpinnings, and recurrent copy number variations together with ATRX and DAXX mutations are a characteristic feature of non-functional PanNETs [12]. However, such alterations might be more prevalent in metastatic insulinomas, of which only few are present in the cohorts previously reported in the literature. Interestingly, in a cohort with multiple rare malignant/metastatic insulinomas, a high number of chromosomal aberrations was strongly associated with metastases [35]. As *ATRX*/*DAXX* mutations and ALT correlate with copy number variations and chromosomal instability [21, 36], this may be a reflection of *ATRX*/*DAXX* mutations that were not tested at that time. Loss of the tumor suppressor *CDKN2A* has been reported once before in a malignant insulinoma [37] and was recently described as a marker of malignant behavior in non-functional PanNET [26].

**Fig. 2:**
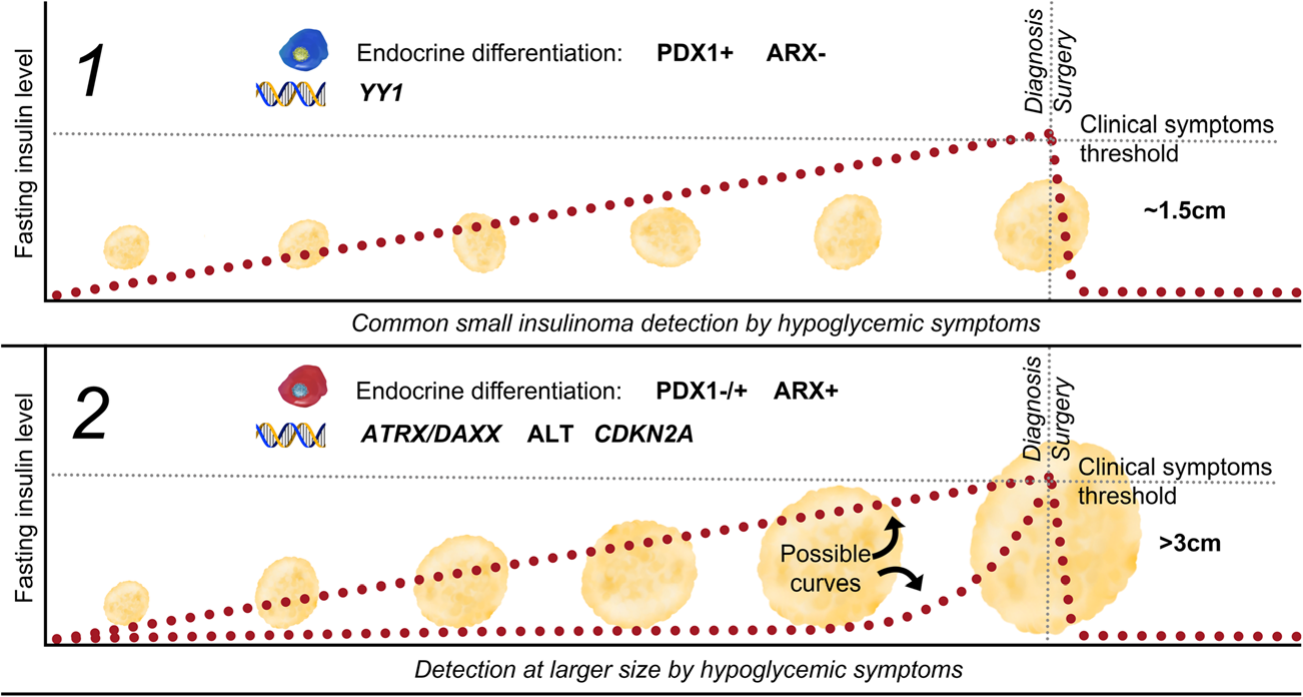
Tumorigenic mechanisms in clinically defined insulinomas. Hypothetic distinct pathways of tumorigenesis in clinical insulinomas based on previously published data in combination with current findings [1, 12, 26, 35]. 1 Typical small insulinomas characterized by recurrent YY1 mutations (25%), neutral, or amplified chromosomal copy numbers, and endocrine transcription factor expression consistent with normal beta-cell differentiation (PDX1+/ARX−). 2 Large insulinomas with distinct tumorigenic mechanisms often seen in non-functional PanNETs and endocrine transcription factor expression inconsistent with normal beta-cell differentiation (ARX+)

Several studies have reported large insulinomas to be malignant more often, and the late symptomatology suggests relatively low or acquired insulin production (Fig. 2) [1, 38, 39]. The ARX transcription factor is not expected to be expressed in insulinomas [40], as it is not expressed in pancreatic beta-cells [41, 42]. In contrast, around 50–60% of non-functional sporadic PanNETs express ARX [27]. Although focal nesidioblastosis was considered as explanation for ARX expression, it was deemed highly unlikely due to the characteristic tumor morphology, random peptide hormone expression, and the presence of metastases [43].

Acquired insulin production could be the result of transdifferentiation of ARX positive non-functional PanNETs or (subclinical) glucagonomas. The latter phenomenon has been shown in mice [44, 45], and a recent report described a human non-functional PanNET that progressed into a metastatic insulinoma with liver metastases over the course of 2 years [46]. Importantly, all malignant cases in this study had hypoglycemic symptoms at presentation, which resolved after surgery of the primary tumor. All but one metachronous liver metastases (patient 2) gave new episodes of symptomatic hypoglycemia when diagnosed. Interestingly, in patient 2, both the primary and the corresponding liver metastasis only had few PDX1-positive cells (potential insulin producing cells), so perhaps the solitary liver metastasis (3 cm) did not grow to a size in which a low percentage of cells could have caused symptoms.

There are several strengths of this study. The assay to assess expression of the nuclear transcription factor ARX is very robust. Whole slides that were stained show no variable expression due to fixation, and pancreatic islets, enteroendocrine duodenal/antrum cells serve as positive internal controls. Although several other markers have been proposed based on molecular analyses, protein, or mRNA expression, this is the first study to show a clear association of one single immunohistochemical marker with malignant behavior in insulinomas [35, 47, 48].

The small cohort size and number of metastatic cases is a limitation of this study, caused by the fact that metastatic insulinomas are very rare. Validation of our findings in other cohorts would be of important value. For the limitations inherent to cut-off scoring systems and TMAs, we have tried to minimize their effect by scoring blinded by two independent observers, confirming key percentages by digital analysis, and staining whole slides to confirm immunohistochemical expression when necessary.

To conclude, large tumor size (> 3 cm) was confirmed to be a strong marker of metastatic behavior in insulinomas. In addition, we found that large metastatic insulinomas are driven by tumorigenic mechanisms often seen in non-functional PanNETs, but not in small indolent insulinomas. Furthermore, we demonstrate that ARX—which is normally not present in beta-cells—is expressed in a subset of insulinomas and is associated with large tumor size, ALT, and metastatic disease. In contrast, ARX expression was absent in any of the small indolent insulinomas in this study or the previous literature. We hypothesize that cellular differentiation and tumorigenic mechanisms more closely related to non-functional PanNETs are important for the development of malignant insulinoma. Our findings with regard to rare metastatic insulinomas may be of value to personalize follow-up and treatment strategies in the future.
